# Achieving High-Performance Polypropylene-Based Synthetic Paper with High-Modulus Organic Oligomer and Biaxial Stretching Force Field

**DOI:** 10.3390/polym17212951

**Published:** 2025-11-05

**Authors:** Zhenkun Wang, Quanjia Du, Weiyouran Hong, Guiying Yu, Haoran Wang, Yanshan Feng, Xinyu Chen, Hongrun Li, Shaoyun Guo, Chunhai Li

**Affiliations:** 1 National Key Laboratory of Advanced Polymer Materials, Polymer Research Institute, Sichuan University, Chengdu 610065, China; 2 Sichuan Provincial Engineering Research Center of Plastic/Rubber Complex Processing Technology, Chengdu 610065, China

**Keywords:** synthetic paper, hydrogenated C9 petroleum resin, biaxial stretching force field, high modulus, polypropylene

## Abstract

The widespread replacement of cellulose paper with polypropylene (PP)-based synthetic paper has been hindered by the relatively low stiffness and modulus of PP. Conventional approaches that incorporate rigid inorganic fillers can enhance the modulus but typically compromise processability and mechanical performance. In this work, we propose a dual strategy by introducing high-modulus organic hydrogenated resin fillers (C9) and applying a biaxial stretching force field. The biaxial stretching process not only promotes PP crystallization but also significantly improves the uniform dispersion of C9 fillers. As a result, a composite paper with ultrafine C9 dispersion and a crystalline self-reinforced structure was successfully fabricated. The composite exhibits a modulus that is 38% higher than that of biaxially stretched neat PP and 218% higher than that of unstretched neat PP. Furthermore, under biaxial stretching, the C9 fillers impart a toughening effect, effectively overcoming the conventional stiffness–toughness trade-off. This work therefore provides a promising strategy for the scalable fabrication of high-performance PP-based synthetic paper.

## 1. Introduction

Paper is extensively used in cultural printing, sanitary products, and many other fields, with global production projected to reach 420 million tons by 2025 [1]. However, this enormous demand raises concerns regarding wood resource depletion and energy consumption [2,3]. To address these challenges, polymer-based synthetic paper has attracted increasing attention [4]. To date, various polymers such as polyethylene (PE) [5], polypropylene (PP) [6,7], and polyvinyl chloride (PVC) [8] have been employed in synthetic paper manufacturing. Among them, PP is the most promising base material owing to its excellent biaxial stretching capability, as well as its combination of low density, chemical resistance, and recyclability [9,10,11]. Nevertheless, the inherent flexibility of PP molecular chains results in insufficient stiffness [12,13,14]. The stiffness of paper, quantified by its modulus, is critical for performance in printing, writing, and high-end packaging applications. However, the modulus of PP-based paper remains only 1–2 GPa, far below the 2–5 GPa of traditional cellulose paper, severely limiting its application [15,16,17]. Therefore, improving the stiffness—i.e., the modulus—of PP-based paper is crucial for enabling its broader application.

A number of strategies have been explored to enhance the modulus of PP, including chemical modification [18,19], physical blending [20,21], and crystal structure regulation [22,23]. Compared with chemical modification, physical blending and crystalline structure regulation are more attractive due to their superior processability, lower cost, and formulation flexibility. In particular, physical blending introduces high-modulus fillers that restrict matrix deformation and facilitate stress transfer within the PP matrix [24,25,26]. The reinforcing effect strongly depends on the filler’s characteristics, such as shape [27], size [28,29], dispersion [30,31], and compatibility [32,33] with PP. Notably, fillers rather than polymers typically serve as the reinforcing phase, thereby enhancing the modulus severalfold [34]. However, conventional inorganic fillers often exhibit poor compatibility with PP, which not only limits modulus enhancement but also deteriorates mechanical properties [34,35,36,37]. In addition, their presence inevitably undermines the processability and biaxial stretchability of PP, both of which are critical for fabricating high-modulus synthetic paper [38,39]. Thus, identifying novel reinforcing fillers is of great importance for developing high-performance PP-based paper.

Hydrogenated C9 petroleum resin is an amorphous oligomer possessing a unique aromatic ring structure and a high glass transition temperature (Tg ≈ 80–140 °C) [40,41]. Below the glass transition temperature, dispersed C9 resin acts as a rigid filler enhancing material modulus; above this temperature, it softens or melts, thereby maintaining the composite material’s melt processability and biaxial tensile properties [42,43]. Such processing properties allow C9 resin to be incorporated into the PP matrix via simple melt blending [44,45,46]. Furthermore, owing to similar solubility parameters, C9 resin exhibits excellent compatibility with PP, resulting in superior dispersion within the PP matrix compared with conventional inorganic fillers [47]. These features suggest that C9 can serve as a novel and effective stiffening agent for manufacturing high-modulus PP-based synthetic paper.

As a semi-crystalline polymer, PP can also achieve modulus enhancement through the construction of a crystalline self-reinforcing structure [48,49]. Such structures are typically induced by external fields, including electric [50], magnetic [51,52], and mechanical force fields [53,54,55]. Among these, the biaxial stretching force field is particularly effective, as it promotes the formation of high-modulus crystals and rigid amorphous fractions (RAF) by overcoming the entropic elasticity of molecular chains [56,57,58,59]. For instance, biaxially oriented polypropylene (BOPP) films exhibit an increase in crystallinity of nearly 30% and a modulus 3–4 times higher than that of unoriented cast films [60,61]. Similarly, biaxial stretching is also the most effective method to fabricate PP-based synthetic paper with enhanced modulus.

Although enhancing the modulus of PP through biaxial stretching is a common strategy, it is scarcely to enhance the modulus of PP by the combination of C9 filler and biaxial stretching. Furthermore, a detailed understanding of such combined synergistic effects is still lacking. To address this problem, this work systematically investigates the synergistic effect of biaxial stretching and C9 filler on the microstructure and properties of PP-based composite paper, and then elucidates the underlying stiffening mechanism. Our results demonstrate that biaxial stretching not only promotes PP crystallization but also improves the uniform dispersion of C9 filler. Simultaneously, C9 filler restricts chain mobility in the amorphous regions, leading to the formation of RAF. The combined effects of crystallinity enhancement and RAF formation result in a significant modulus improvement. Moreover, under biaxial stretching, C9 filler exhibits a unique toughening effect, thereby overcoming the conventional stiffness–toughness trade-off. This work, therefore, provides a promising strategy for the scalable fabrication of high-performance PP-based synthetic paper.

## 2. Materials and Methods

### 2.1. Materials

Commercially available polypropylene (PP, trade name 2020s), obtained from Formosa Plastics Co., Ltd. (Taipei, China), has a melt index of 3.0 g/10 min (230 °C, 2.16 kg). The hydrogenated C9 petroleum resin (trade name HP140) was supplied by Henghe Materials Technology Co., Ltd. (Ningbo, China), with a glass transition temperature (Tg) of 92.5 °C (measured by DSC, Q20, TA Instruments, Newcastle, DE, USA). The structure of hydrogenated C9 petroleum resin is shown in Figure S1b.

### 2.2. Sample Preparation

#### 2.2.1. Preparation of PP/C9 Composite Sheets

The PP/C9 composite particles were produced using a twin-screw extruder (SHJ-20, Nanjing Jiantai Electromechanical Co., Ltd, Nanjing, China). The processing temperature was set at 195 °C, 205 °C, 210 °C, and 205 °C, from the hopper to the die. The C9 filler was incorporated into the composite particles at contents of 3%, 5%, 10%, and 15%. Afterward, these particles were hot-compressed using a plate vulcanizer (Ky3201s, Kaiyan Precision Machinery Equipment Factory, Shanghai, China) at a temperature of 215 °C and a pressure of 15 MPa. The final PP/C9 composite sheets, measuring 100 mm in length, 100 mm in width, and 1 mm in thickness, were obtained.

#### 2.2.2. Preparation of PP/C9 Composite Paper Through the Biaxial Stretching

**Figure 1:**
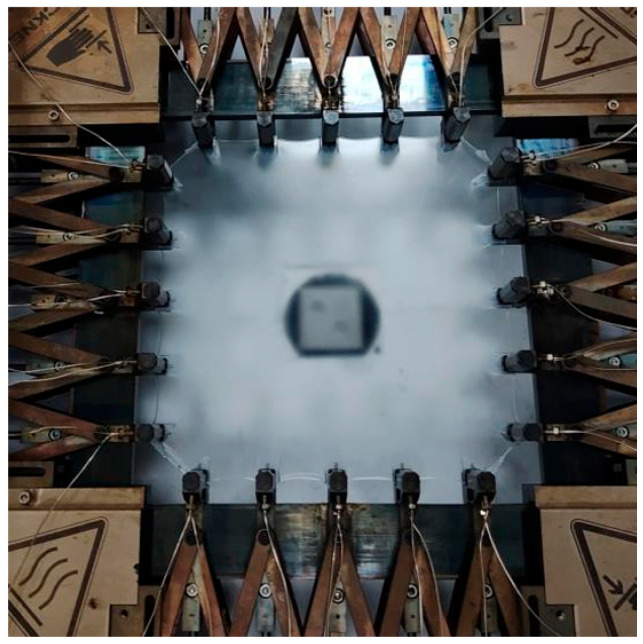
A schematic diagram of the biaxial stretching process for preparing the PP/C9 composite paper.

As shown in Figure 1, the PP-based paper was prepared by simultaneously biaxially stretching the corresponding sheets in the biaxial stretching machine (Machine Karo IV, Brückner, Siegstetten, Germany). The preheating temperature was 160 °C for 3 min, and the stretching temperature was 130 °C. The stretching ratios of 3 × 3, 5 × 5, and 7 × 7 were applied with a deformation rate was 10%/s. The sample here is designated as C9-X-Y, where X represents the content of C9 filler, and Y denotes the biaxial stretching ratio.

### 2.3. Characterization

#### 2.3.1. Morphology and Dispersion Evolution of C9 Filler

**Scanning electronic microscopy (SEM):** SEM analysis was performed to investigate both the evolution of morphology and distribution of the C9 filler under the biaxial stretching force field and the toughening mechanism of the C9 filler on the PP matrix by observing its plastic deformation. To achieve these purposes, these samples were first cooled in liquid nitrogen for 8 h and then fractured along a pre-made notch. Afterward, the fracture surfaces were coated with a thin gold layer via sputter deposition and analyzed using a scanning electron microscope (SEM, JSM-5900LV, JEOL, Akishima-shi, Japan). The acceleration voltage was set at 10 kV.

#### 2.3.2. Crystalline Information

**Polarized optical microscopy (POM):** To observe the crystalline morphology within composite paper, the thin slice (about 30 µm thick) was prepared from the samples using a microtome along the thickness direction. Then, this thin slice was placed between two glass slides and observed under a polarized optical microscope (POM, BX51, OLYMPUS, Tokyo, Japan).

**Differential scanning calorimeter (DSC):** Thermal analysis was carried out using a DSC (Q20, TA Instruments, Newcastle, DE, USA) under a nitrogen atmosphere. The sample (6–10 mg) was initially equilibrated at −40 °C. Subsequently, the sample was heated to 230 °C at a rate of 10 °C/min, held for 3 min to eliminate prior thermal effects, followed by cooling to −40 °C at the same heating rate. Finally, the sample was reheated to 230 °C at 10 °C/min. The crystallization behavior was analyzed by recording the first heating and cooling curves. The crystallinity ($X_{c}$) was calculated using Equation (1):

(1) $$X_{c}=\frac{∆H_{m}}{W\cdot {∆H}_{m}^{0}}$$

where the $∆H_{m}$ is the melting enthalpy of PP in each sample, the $∆H_{m}^{0}$ is the melting enthalpy of PP at 100% crystallinity (approximately 209 J/g) [62], and W represents the weight fraction of PP in each sample.

**Two-Dimensional Small-Angle X-ray Scattering (2D-SAXS):** 2D-SAXS measurements were performed with a Xeuss 2.0 system (Xenocs, Grenoble, France), where the sample-to-detector distance was set to 2500 mm, and the scattering vector (q) ranged from 0.008 to 0.183 nm^−1^. Scattering data were captured using a Pilatus 300K detector (Dectris, Disentis, Switzerland; 680 × 600 pixels, pixel size = 172 µm). The beam center and sample-to-detector distance were calibrated using a silver behenate standard. The 1D-SAXS curves were obtained by integrating the corresponding 2D-SAXS patterns.

**Two-dimensional wide-angle X-ray Diffraction (2D-WAXD):** 2D-WAXD was performed on the same Xeuss 2.0 system (Xenocs, Grenoble, France) with a sample-to-detector distance of 172 mm. The system parameters were also calibrated using a silver behenate standard. The 1D-WAXD curve was obtained by integrating the 2D-WAXD pattern.

#### 2.3.3. Mechanical Test

**Dynamic Mechanical Analysis Testing (DMA):** The dynamic mechanical properties of PP/C9 composite paper were measured using a dynamic mechanical analyzer (Q800, TA Instruments, Newcastle, DE, USA). Rectangular samples (20 mm × 5 mm) were tested in tensile mode. The frequency was set to 1 Hz and the amplitude to 20 µm. The test temperature range was set from −50 °C to 150 °C, with a heating rate of 5 °C/min. Five parallel samples of each composite paper were tested, and then the average values with standard deviations were calculated.

**Tensile Test:** Tensile tests were conducted at ambient temperature (23 °C) using a universal testing machine (Instron 68TM-10, Boston, MA, USA) on dumbbell-shaped samples, with a crosshead speed of 100 mm/min. Eight parallel samples of each composite paper were tested, and the average values with standard deviations were calculated.

**Tear Test:** Tear tests were also conducted at ambient temperature (23 °C) using a universal testing machine (Instron 68TM-10, Boston, MA, USA) in accordance with ASTM D1004-21. The crescent-shaped samples, as shown in Figure S2, were tested at a crosshead speed of 50 mm/min. The tear strength was calculated using Equation (2), and the tear toughness was determined by integrating the load–displacement curve. To eliminate the effect of thickness variations, the tear toughness was normalized by the sample thickness, and the result was reported as tear energy. Eight parallel samples of each composite paper were tested, and the average values with standard deviations were calculated.

(2) $$T_{s}=\frac{F}{d}$$

where $T_{s}$ is the tear strength (kN/m), F is the maximal tearing force (N), and d is the thickness of the sample (mm) [63].

#### 2.3.4. Toughening Mechanism

**Super-depth-of-field digital microscopy:** To investigate the toughening mechanism of the C9 filler after biaxial stretching, the fracture surfaces of tensile-tested samples were recorded by a super-depth-of-field digital microscope (Keyence VHX-1000C, Osaka, Japan) at a magnification of 100×.

## 3. Results and Discussion

### 3.1. Morphology and Dispersion Evolution of C9 Filler

**Figure 2:**
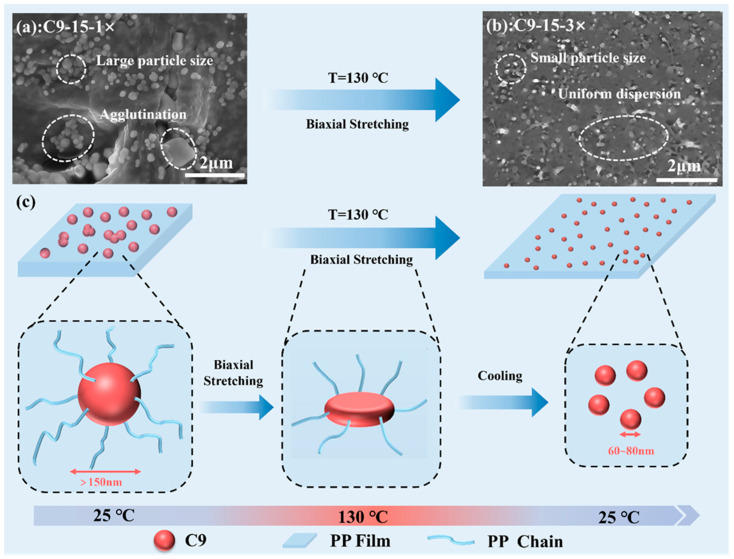
The dispersion morphology of C9 and its evolutionary process under the biaxial stretching force field; (**a**) the SEM images showing the agglutination for PP/C9 composite paper with 15% C9 before biaxial stretching; (**b**) the SEM images showing the uniform dispersion of PP/C9 composite paper with 15% C9 after 3 × 3 biaxial stretching; (**c**) the schematic showing the evolutionary of process of C9 under the biaxial stretching force field.

The morphology and dispersion of the C9 filler determine the performance of the PP/C9 composite paper, which is hence studied first in Figure 2. For simplicity, taking the PP-based composite paper with the highest C9 loading as an example, illustrates the dispersion of C9 under the biaxial stretching force field. Figure 2a shows the poor dispersion of the C9 filler, i.e., its agglomeration with the sizes exceeding 150 nm in the unstretched samples. Notably, after the biaxial stretching ratio of 3 × 3 at 130 °C, the dispersion of C9 filler is improved, whose size is dramatically reduced to 60–80 nm (Figure 2b). This morphological evolution and improved dispersion of the C9 filler can be attributed to the synergistic deformation of the PP/C9 composite under the biaxial stretching force field. From the structural perspective, both PP and hydrogenated C9 petroleum resin are nonpolar materials composed solely of carbon and hydrogen, which grants them strong mutual interaction and excellent compatibility (Figure S1). At the biaxial stretching temperature of 130 °C, the C9 filler (Tg ≈ 92.5 °C) softens significantly. Under the biaxial stretching force field, the orienting PP chains effectively exert peeling and shear forces upon the softened C9 filler, reducing its domain size and enhancing its dispersion uniformity (Figure 2c). The crystallization behavior and mechanical properties may be improved by the uniform dispersion of C9 filler, which will be discussed later.

### 3.2. Crystallization Behavior of PP/C9 Composite Paper

**Figure 3:**
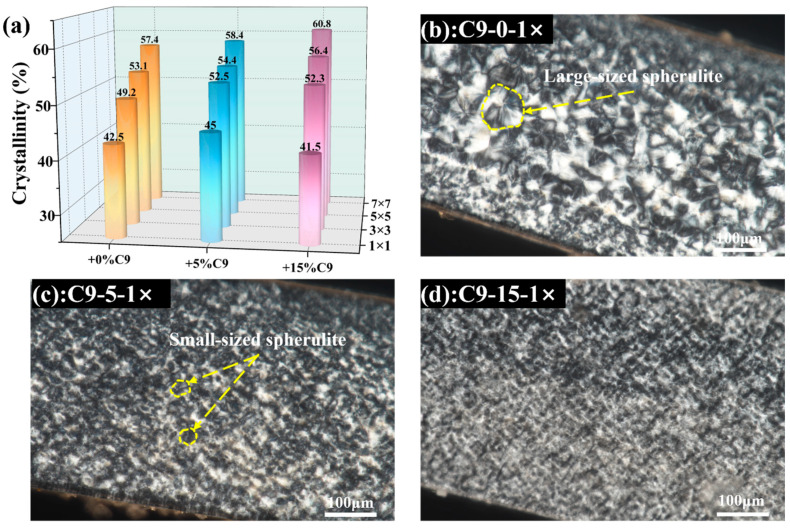
Crystallization information for PP/C9 composite paper: (**a**) crystallinity variation when increasing the biaxial stretching ratio and C9 content; (**b**–**d**) the POM images showing the crystalline size for the composites before stretching.

As mentioned earlier, the crystallization behavior of PP/C9 composite paper may be influenced by the uniformly dispersed C9 filler. Hence, the crystallization information of the PP/C9 composite paper is studied. The DSC first heating curves for composite paper with different C9 contents and biaxial stretching ratios are shown in Figure S3. Notably, the C9 filler is the amorphous oligomer lacking crystallization capability, as evidenced by the absence of both a melting peak in the heating curve and a crystallization peak in the cooling curve compared with PP in Figure S4. Hence, the melting peak on the DSC first heating curves should be attributed to the PP. As depicted in Figure 3a, the crystallinity is governed by the biaxial stretching force field and the C9 filler. The biaxial stretching force field here has a positive effect on the crystallinity. Specifically, when the C9 content is fixed, the crystallinity rises as the biaxial stretching ratios increase due to the stretch-induced crystallization [64,65]. On the contrary, increasing the C9 content under the different biaxial stretching ratios results in varying crystallinity trends. When the biaxial stretching ratio is kept constant at 1 × 1 and 3 × 3, increasing the C9 content from 0% to 15% first increases the crystallinity and then decreases it. At higher biaxial stretching ratios (5 × 5 and 7 × 7), increasing the C9 content consistently increases the crystallinity. Here, the reasons for changes in crystallinity can be attributed to the nucleation effect of the C9 filler. Based on Section 3.1 Morphology and Dispersion Evolution of C9 Filler, at lower biaxial stretching ratios like 1 × 1 and 3 × 3, the agglomerates of higher C9 content may hardly be broken, due to the weak synergistic deformation caused by the poor stretching force. The nucleation effect of the C9 filler here is restricted. Specifically, under a lower biaxial stretching ratio, when the C9 content increases to 5%, the C9 agglomerates can be broken and dispersed in the PP matrix, increasing the crystallinity. When the C9 content increases to 15%, the C9 agglomerates are hardly broken, thus decreasing the crystallinity. Conversely, at high biaxial stretching ratios like 5 × 5 and 7 × 7, the C9 agglomerates are easily broken by the synergistic deformation of the oriented PP molecular chains, which can maximize the potential of the nucleation effect of C9 filler. As a result, increasing the C9 content increases the crystallinity.

To further clarify the nucleation effect of the C9 filler, the crystalline morphology of the PP/C9 composite paper under the biaxial stretching ratio of 1 × 1 is discussed in Figure 3b. For the PP paper without adding the C9, the large spherulites of the PP are observed in Figure 3b When the C9 content increases to 5%, the size of the spherulites significantly decreases (Figure 3c). Further increasing the C9 content to 15%, the size of the spherulite becomes smaller (Figure 3d). This evolution in crystalline morphology can be attributed to the C9 filler acting as a heterogeneous nucleating agent, which increases the number of crystalline nuclei and refines the spherulite size of PP [42].

**Figure 4:**
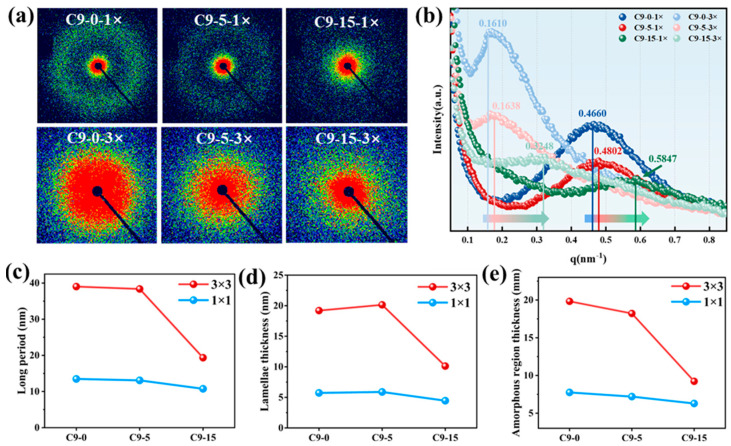
The 2D-SAXS patterns of PP/C9 composite paper with different C9 content and stretching ratios: (**a**) the 2D-SAXS scattering patterns; (**b**) corresponding 1D-SAXS scattering curves; (**c**) the long period (Lp); (**d**) the lamellae thickness (Lc), and (**e**) interlamellar amorphous region thickness (L_a_).

The 2D-SAXS patterns of the PP/C9 composite paper are presented in Figure 4a. To simplify, this study selectively exhibits the 2D-SAXS patterns for samples with stretching ratios of 1 × 1 and 3 × 3. In Figure 4a, all samples exhibit the scattering circle, which indicates these samples form a random microstructure. To quantitatively illustrate the effect of both C9 filler and biaxial stretching force field on the structure, the structure parameters of PP crystals are calculated. As depicted in Figure 4b–e, when the C9 content is kept constant, an increase in the biaxial stretching ratio leads to an increase in the long period (Lp), lamellar thickness (Lc), and interlamellar amorphous region thickness (La), which may be due to the stretch-induced crystallization. On the contrary, the structure parameters exhibit varying trends with the increase in C9 content at a fixed stretching ratio. With the stretching ratio maintained at 1 × 1, the Lp, Lc, and La remain almost constant. When the stretching ratio is kept at 3 × 3 while increasing the C9 content, the Lp decreases, the Lc decreases, and the interlamellar amorphous region thickness also decreases, which indicates that increasing C9 content leads to smaller crystals of PP.

**Figure 5:**
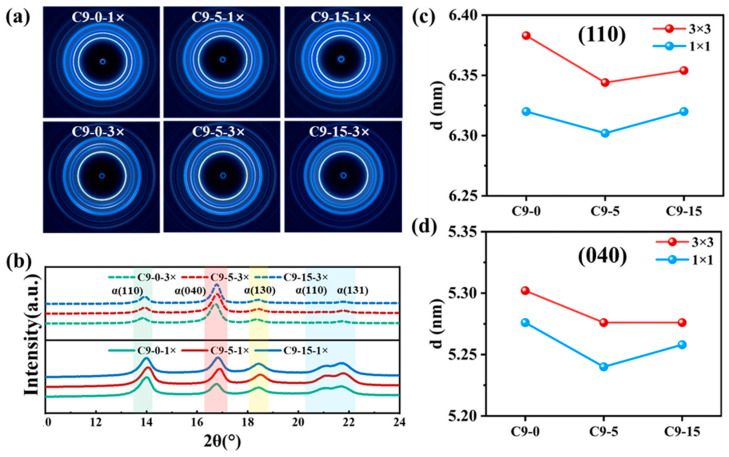
The 2D-WAXD patterns of PP/C9 composite paper with different C9 content and stretching ratios: (**a**) the 2D-WAXD scattering patterns; (**b**) the corresponding 1D-WAXD scattering curves; (**c**) and (**d**) the corresponding distance of interplanar crystal (110) and (040).

Figure 5a presents the 2D-WAXD pattern of the PP/C9 composite paper. The varying radii of the “Debye-Scherrer diffraction circle” on different lattice planes also indicate that the crystals within the composite paper are randomly distributed, exhibiting no obvious orientation. This phenomenon may be attributed to the same stretching ratio being applied in both the longitudinal and transverse directions. The corresponding interplanar crystal distances are also calculated according to the 1D-WAXD curves (Figure 5b–d). When the stretching ratio remains constant, the distance of interplanar crystal (110) and (040) first reduces and then increases as the C9 content increases from 0% to 15%. This is because the low-content C9 filler is directly embedded between crystal regions, physically expanding the interplanar crystal distance. When the C9 content increases, it causes the heterogeneous nucleation to increase the density of the crystal nucleus, thereby reducing the interplanar crystal distance. When the C9 content is kept constant, increasing the stretching ratio increases the distance between the interplanar crystal (110) and (040), because the molecular chains between crystalline regions are oriented under the stretching force field.

### 3.3. Mechanical Properties of PP/C9 Composite Paper

Mechanical properties are key indicators for the polymer-based synthetic paper. Hence, the effects of C9 filler and biaxial stretching force field on mechanical properties are systematically investigated.

**Figure 6:**
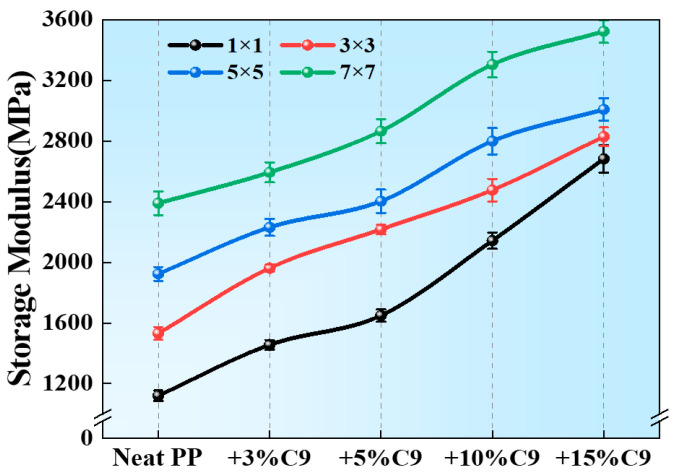
The storage modulus at 25 °C of PP/C9 composite paper with different C9 contents and biaxial stretching ratios.

In this work, the storage modulus at 25 °C (E′_25_) is used to study the influence of C9 content and biaxial stretching force field on the modulus of the PP/C9 composite paper. As shown in Figure 6, increasing the content of C9 significantly enhances the E′_25_ at the constant stretching ratio. In addition, the E′_25_ also rises with the increase in stretching ratios. When the C9 content reaches 15% and the stretching ratio is 7, the enhancement ratio of E′_25_ reaches 218% compared with the unstretched PP without C9. These results demonstrate a dual effect between the biaxial stretching force field and C9 filler in enhancing the modulus of the PP/C9 composite paper. To clarify the underlying mechanism for this enhancement, the structure evolution and molecular mobility are further studied through the loss modulus (E′′) and the loss factor (tanδ).

**Figure 7:**
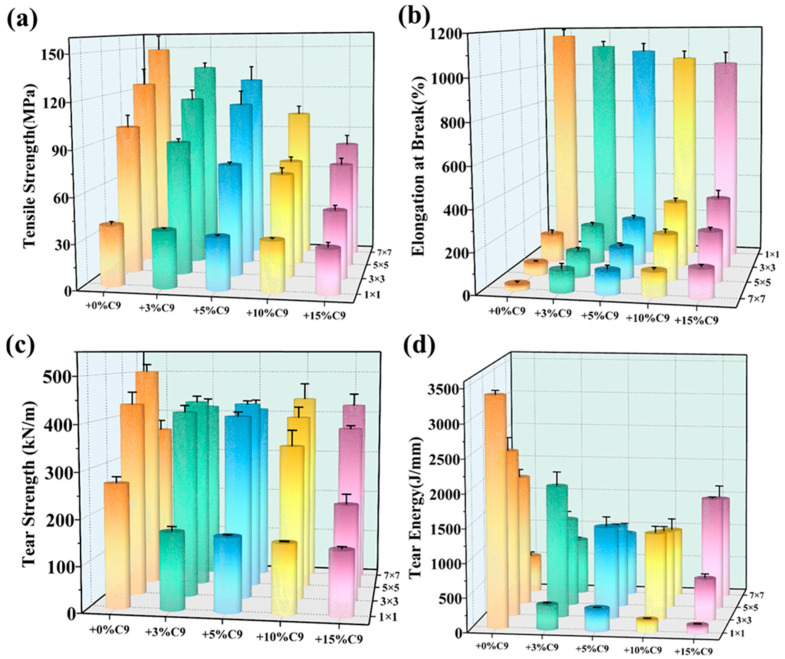
The mechanical properties of PP/C9 composite paper with different C9 content and stretching ratio: (**a**) the tensile strength; (**b**) the elongation at break; (**c**) the tear strength; and (**d**) the tear energy.

Figure 7a illustrates the synergistic effect of C9 content and biaxial stretching force field on the tensile strength of PP/C9 composite paper. When the biaxial stretching ratio remains constant, increasing the C9 content reduces tensile strength, which can be attributed to the C9 aggregates acting as the stress concentration point, thereby accelerating material failure. At a constant C9 content, tensile strength significantly improves with a higher stretching ratio. This enhancement is because the molecular chain is highly oriented under the biaxial stretching force field, increasing the proportion of covalent bonds in the load direction. In contrast to tensile strength, the elongation at break of PP/C9 composite paper decreases significantly with increasing biaxial stretching ratio (Figure 7b). The reason for this is that the molecular conformation transitions from random coils to oriented chains under the biaxial stretching force field, thereby reducing their deformation capacity [66]. On the contrary, increasing the C9 content under the fixed biaxial stretching ratio results in different change trends. When the biaxial stretching ratio is kept constant at 1 × 1, the elongation at break reduces as the C9 content increases from 0% to 15%, due to the stress concentration effect of the C9 filler. When the biaxial stretching ratio is kept constant at 3 × 3, 5 × 5, and 7 × 7, the elongation at break increases with higher C9 content. This can be attributed to the impact of the biaxial stretching on the morphology and dispersion of the C9 filler. Specifically, at higher biaxial stretching ratios, the stress concentration effect of C9 is alleviated, due to its finer particle size and more uniform dispersion (Figure 2b).

Different from tensile strength mentioned above, the tear strength reflects the ability of synthetic paper to resist crack propagation. Figure 7c shows intense dependency of tear strength on C9 content and biaxial stretching force field. When the stretching ratio is kept constant at 1 × 1, 3 × 3, and 5 × 5, increasing the C9 content reduces tear strength. This may be attributed to the fact that the crack is difficult to resist for the C9 with low molecular weight. On the contrary, increasing the biaxial stretching ratio exhibits a different trend in tear strength. At a fixed C9 content below 5%, the tear strength first rises and then decreases with increasing biaxial stretching ratio. When the C9 content is kept constant at 10% and 15%, increasing the stretching ratio increases the tear strength. This may be explained by the fact that the molecular chain is more difficult to break due to the movement of molecular chains restricted by a more uniformly dispersed C9 filler. As shown in Figure 7d, the tear energy of PP/C9 composite paper is also influenced by the C9 content and biaxial stretching force field. Under different biaxial stretching ratios, increasing the C9 content results in different trends of tear energy. When the stretching ratios are constant at 1 × 1 and 3 × 3, increasing C9 content decreases the tear energy. As the stretching ratio increases to 5 × 5, the tear energy shifts from the before rapid reduction to the initial decrease and then increase. Further increasing the stretching ratio to 7 × 7, the tear energy increases with rising C9 content. Similarly, increasing the stretching ratios at different C9 contents also causes different tear energy trends. For the PP without C9, increasing the stretching ratio decreases the tear energy. Fixing the C9 content at 3%, 5%, and 10%, the tear energy first rises and then reduces as the stretching ratio increases. As the C9 content further increased to 15%, the tear energy continued to rise with increasing stretching ratio. This may be attributed to the fact that the crack propagation pattern relies on the dispersion and particle size of the C9 filler under the biaxial stretching force field. As the crack propagates, the paths become more zigzagged due to the more uniform dispersion of the C9 filler, which ultimately enhances the tear energy. It is also noted that the particle size of C9 gradually decreases as the biaxial stretching ratio increases. At higher biaxial stretching ratios, the particle size of C9 is so small that it cannot restrict crack propagation. Consequently, the tear energy of the composite paper with lower C9 content (like 3% and 5%) inevitably decreases.

### 3.4. Modulus Enhancement Mechanisms of PP/C9 Composite Paper

**Figure 8:**
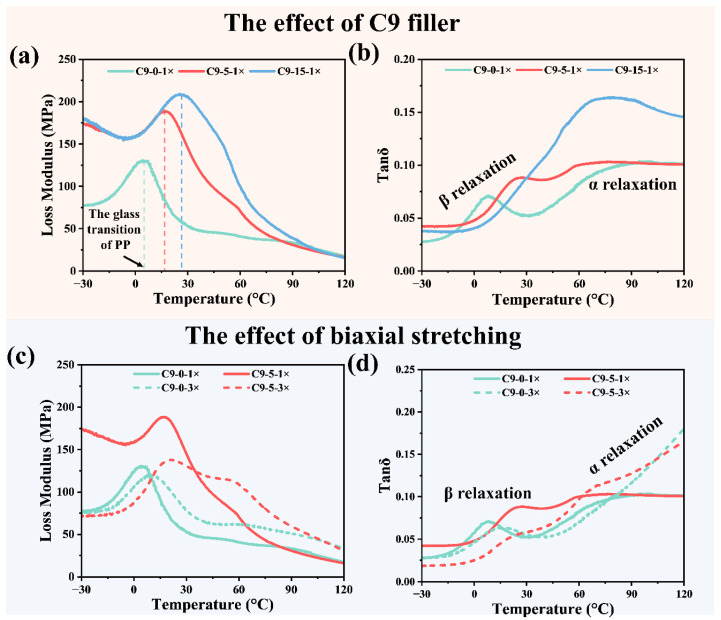
The dynamic mechanical properties of PP/C9 composite paper with different C9 contents and stretching ratios: (**a**) the loss modulus–temperature curve of unstretched composite paper containing 0%, 5%, and 15% C9; (**b**) the loss factor–temperature curve of unstretched composite paper containing 0%, 5%, and 15% C9; (**c**) the loss modulus–temperature curve of the unstretched composite paper containing 0% and 5% C9 with the stretch ratio of 1 × 1 and 3 × 3 and (**d**) the loss factor-temperature curve of the composite paper containing 0% and 5% C9 with the stretch ratio of 1 × 1 and 3 × 3.

To investigate the mechanism of enhanced modulus by C9 filler and biaxial stretching force field, the loss modulus (E′′) and the loss factor (tanδ) of the unstretched composite paper containing 0%, 5%, and 15% C9 filler are selected for analysis. As illustrated in the E′′-temperature curve (Figure 8a), the peak of E′′ shifts to higher temperatures with an increase in C9 content. This peak of E′′ is associated that increasing the C9 content enhances the Tg of PP/C9 composite paper, indirectly confirming its reinforcing effect on the modulus.

Beyond the E′′, the tanδ-temperature curve is also studied to obtain more information about the effect of C9 filler (Figure 8b). For the PP without C9, the tanδ-temperature curve shows peaks in both the low- and high-temperature regions. The tanδ peak in the low-temperature region corresponds to the β-relaxation peak, which is associated with the glass transition of PP, and the tanδ peak in the high-temperature region corresponds to the α-relaxation peak, associated with the rigid amorphous fraction (RAF). The movement of molecular chains within the RAF is constrained by the crystalline region, and thus relaxation only occurs at higher temperatures. As depicted in Figure 8b, an increase in C9 content results in the β-relaxation peak shifting to higher temperatures. When the content of C9 reaches 15%, the β-relaxation peak merges with the α-relaxation peak [67,68,69]. This merging results in a single peak covering a wider temperature range with a higher peak value. This series of results indicates that C9 exhibits excellent compatibility with the PP matrix. The mechanism of enhanced modulus can be attributed to the C9 filler restricts the relaxation behavior of chain segments within the amorphous region, transforming the amorphous region into the RAF. As a result, the modulus of composite paper at ambient temperatures is improved.

To investigate the mechanism of the modulus enhancement by the biaxial stretching force field, the E′′ and tanδ of the following four composite papers were selected for analysis, including the unstretched composite paper with 0% and 5% C9 filler, and the composite paper with a stretching ratio of 3 × 3, containing 0% and 5% C9 filler, respectively. As shown in the E′′-temperature curve (Figure 8c), the peak of E′′ shifts to a higher temperature as the stretching ratio increases, which indicates the biaxial stretching force field raises the Tg of PP. Conversely, increasing the stretching ratio reduces the peak value of E′′, meaning a smaller proportion of molecular chains undergo glass transition. The reason for these can be attributed to the fact that the biaxial stretching force field induces orderly alignment of molecular chains, facilitating their incorporation into the crystal. As a result, the proportion of the crystalline regions increases, while that of the amorphous regions decreases. In addition, the mobility of the chain segment is also restricted by the crystalline regions, leading to a higher Tg.

From the loss factor–temperature curve presented in Figure 8d, it can be seen that increasing the stretching ratio raises the temperature of the β-relaxation peak, while reducing its peak value. This is also because the biaxial stretching force field reduces the proportion of molecular chains in the amorphous regions and restricts their mobility. As for the α-relaxation at high temperatures, both the temperature and peak value increase as the biaxial stretching ratio increases. This can be attributed to the biaxial stretching force field enhances crystallinity and increases the lamellar thickness. This results in more molecular chains in the amorphous regions being constrained by crystalline regions, forming RAF. Hence, the α-relaxation peak exhibits a higher value.

In conclusion, the synergistic enhancement of the modulus resulting from the addition of C9 filler and the application of a biaxial stretching force field can be attributed to the following mechanisms. The C9 filler restricts the molecular chain mobility in the amorphous regions and promotes the formation of RAF. Importantly, the biaxial stretching further increases crystallinity and RAF. The combination of an increase in crystallinity and RAF proportion achieves a significant improvement in the modulus of the PP/C9 composite paper.

### 3.5. Toughening Mechanisms of PP/C9 Composite Paper

**Figure 9:**
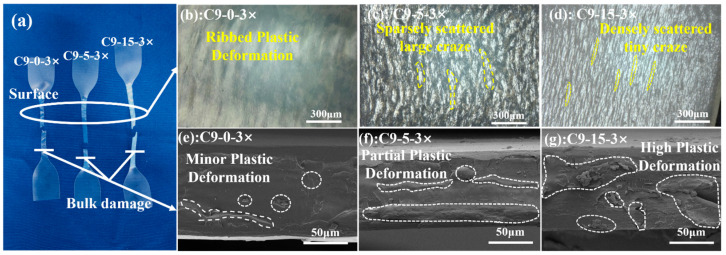
Tensile test fracture behaviors of PP/C9 composite paper: (**a**) fractured samples of C9-0-3×, C9-5-3×, and C9-15-3× after tensile testing; (**b**–**d**) the surface of each of the fractured samples; (**e**–**g**) the bulk damage of each sample.

Mechanical performance of the PP/C9 composite paper exhibits superior toughness; thus, the corresponding mechanism should be studied. Figure 9a shows that increasing the content of C9 filler enhances the stress whitening phenomenon of the tensile samples. As shown in Figure 9b–d, different surface morphologies of the tensile samples with increasing C9 content under the biaxial stretching ratio of 3 × 3 are observed by the super-depth-of-field microscope. Figure 9b shows that the PP without C9 does not exhibit craze, only presenting the ribbed plastic deformation. As the C9 content increases to 5%, the sparsely scattered large craze can be observed (Figure 9c). When the C9 content reaches 15%, the craze grows into the smaller and denser craze (Figure 9d). To further characterize the microstructure of tensile samples, the bulk damage beneath the fracture surface is shown in Figure 9e–g. As shown in Figure 9e, there are a few regions of strip-like and block-like plastic deformation in the PP paper without C9. When the content of C9 increases to 5%, the plastic deformation regions evolve to the longer strip-like and larger block-like region in the PP/C9 composite paper (Figure 9f). Further increasing the C9 content to 15%, the proportion of the plastic deformation regions significantly increases (Figure 9g). Therefore, the reason for the superior toughness of the PP/C9 composite paper is that increasing the C9 content can promote the plastic deformation of the composite paper to form massive crazes.

## 4. Conclusions

In this work, organic rigid fillers and biaxial stretching were employed to fabricate high-modulus polypropylene (PP) composite papers. A series of PP/C9 composite papers with varying C9 contents and biaxial stretching ratios was successfully prepared. The key findings can be summarized as follows: (1) the biaxial stretching field not only promoted the crystallization of PP but also enhanced the dispersion uniformity of the C9 filler; (2) the introduction of C9 restricted the mobility of molecular chains in the amorphous phase, thereby generating rigid amorphous fractions (RAF); (3) the combined effects of the high modulus of C9, the enhanced crystallinity, and RAF formation led to a remarkable increase in modulus—38% higher than that of biaxially stretched neat PP and 218% higher than that of unstretched PP; (4) the PP/C9 composite papers exhibited an 52% improvement in toughness compared with biaxially stretched neat PP without C9. Collectively, these results demonstrate that this work successfully overcomes the classical trade-off between modulus and toughness, simultaneously enhancing both properties in PP-based composite papers, and providing a promising route for the development of high-performance PP synthetic paper.

## Data Availability

The original contributions presented in this study are included in the article/Supplementary Material. Further inquiries can be directed to the corresponding author.

## Supplementary Materials

The following supporting information can be downloaded at: https://www.mdpi.com/article/10.3390/polym17212951/s1; Figure S1: The structure of PP and hydrogenated C9 petroleum resin; Figure S2: The schematic diagram of the geometric dimensions of the tear test sample; Figure S3: The first heating curves for PP/C9 composite paper with different C9 contents and stretching ratios; Figure S4: The heating and cooling curves of C9 filler and neat PP obtained by DSC.
