# Supplementary material for: Achieving High-Performance Polypropylene-Based Synthetic Paper with High-Modulus Organic Oligomer and Biaxial Stretching Force Field
Source: Polymers (Basel). 2025 Nov 5;17(21):2951. doi: 10.3390/polym17212951 (PMC12608191; doi:10.3390/polym17212951)
Supplement: Supplementary file 1 [file polymers-17-02951-s001.zip › polymers-3928386-supplementary.pdf]

Achieving high-performance polypropylene-based synthetic paper with high-modulus organic oligomer and biaxial stretching force field

2. Experiment Section

2.1 Materials

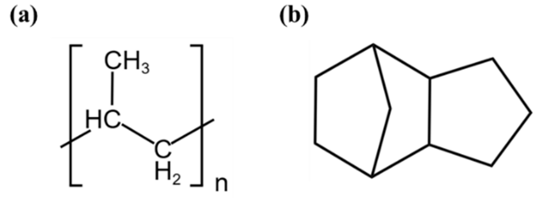

Figure S1. (a) The structure of PP; (b) the structure of hydrogenated C9 petroleum resin.

2.3. Characterization

2.3.3 Mechanical Test

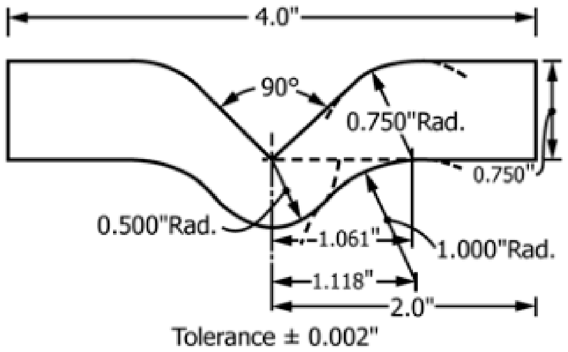

Figure S2. Schematic diagram of the geometric dimensions of the tear test sample.

3. Results and Discussion

3.2 Crystallization Behavior of PP/C9 Composite Paper

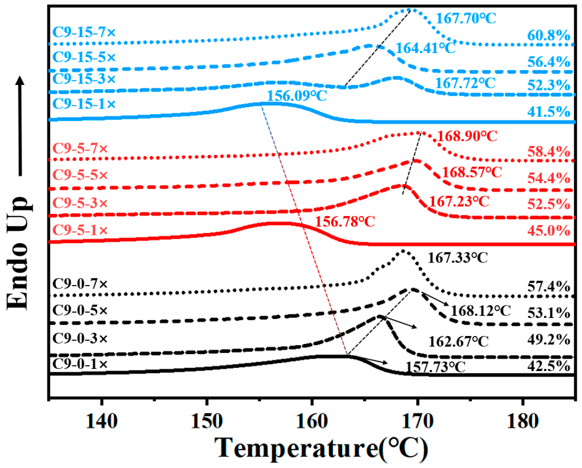

Figure S3. The first heating curves for PP/C9 composite paper with different C9 contents and stretching ratios.

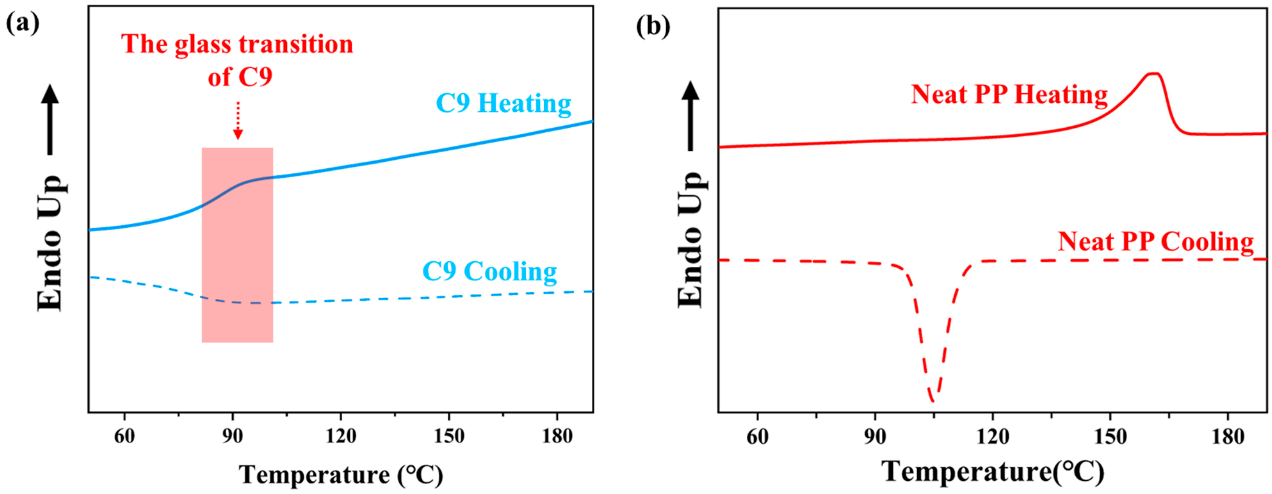

Figure S4. The heating and cooling curves of C9 filler and neat PP obtained by DSC.
